# Evolutionary biology at BGRS\SB-2016

**DOI:** 10.1186/s12862-016-0869-8

**Published:** 2017-02-07

**Authors:** Ancha V. Baranova, Yuriy L. Orlov

**Affiliations:** 1 School of Systems Biology, George Mason University, Fairfax, VA 22030 USA; 2 Research Centre for Medical Genetics, Moskvorechie, 1, Moscow, Russia; 3 Institute of Cytology and Genetics SB RAS, Lavrentyeva, 10, 630090 Novosibirsk, Russia; 4 Novosibirsk State University, Pirogova, 2, 630090 Novosibirsk, Russia

This special BMC Evolutionary Biology BGRS\SB-2016 issue is an integral part of the collection of manuscripts presented at “Bioinformatics of Genome Regulation and Structure\Systems Biology” (BGRS\SB-2016) conference which took place at August 29 - September 2, 2016 in Novosibirsk, Russia. Other issues in this collection include BMC Genetics [1], BMC Plant Biology [2], BMC Genomics [3], BMC Bioinformatics and BMC Systems Biology releases.

International Conference BGRS\SB-2016 (http://conf.bionet.nsc.ru/bgrssb2016/) is 10th installment of the biannual BGRS event which has a long history starting from 1998 in Novosibirsk. The conference organized by Institute of Cytology and Genetics of Siberian Branch of the Russian Academy of Sciences (ICG SB RAS) has focuses on evolutionary biology, systems biology and bioinformatics, gene network analysis, post-genomics and sequencing technologies. Since 1998, BGRS series has grown to large international multi-conference forum where medical doctors, mathematicians, biologists could share their recent theoretical and experimental insights.

In 2016, the BGRS included several parallel events and symposia: the international Symposium “Systems Biology and Biomedicine” (SBioMed-2016) (http://conf.bionet.nsc.ru/ishg2016/en/), Symposium “Cognitive Sciences, Genomics and Bioinformatics” (CSGB-2016) (http://physiol.ru/csgb2016/), and the Second International Conference on the Mathematical Modeling and High-Performance Computing in Bioinformatics, Biomedicine and Biotechnology (MM-HPC-BBB-2016) (http://conf.bionet.nsc.ru/mm-hpc-bbb-2016/en/).

Since 2014, The BGRS Program Committee collaborates with BioMed Central on full-text thematic issues of relevant BMC journals. In recent years, BioMed Central had published several special issues based on best materials presented at the conference, including ones in BMC Genomics (http://www.biomedcentral.com/bmcgenomics/supplements/15/S12), BMC Genetics [4], BMC Evolutionary Biology (http://www.biomedcentral.com/bmcevolbiol/supplements/15/S1), and BMC Systems Biology (http://www.biomedcentral.com/bmcsystbiol/supplements/9/S2).

Additionally, special issues on bioinformatics were published at the “Journal of Bioinformatics and Computational Biology” [5] and “Vavilov Journal of Selection and Breeding” (http://vavilov.elpub.ru/jour/) (in Russian). This year, a special issue in “Molecular Biology” (Springer) was added to the list (http://www.springer.com/life+sciences/journal/11008).

Current issue of BMC Genomics presents reports on genome-wide studies in computational biology discussed at the BGRS\SB-2016 conference.

The paper by Oleg Balanovsky et al. [6] opens this special issue by presenting the results of combined efforts of population geneticists and genetic genealogists in reconstructing phylogeography of human haplogroup Q3. In addition to important conclusions about history of human migrations, this study provides positive example of collaboration between academic and citizen science and demonstrates possibility of well-balanced and careful interpretation of the paternal-side history of human populations.

The work of Vladimir Babenko and colleagues [7] utilizes high resolution map of human genome for genome-wide study of CpG islands and Alu-repeats. Authors demonstrated that the landscapes of these elements are related to chromatin accessibility. As particular example, authors analyzed CpG-rich genome of dog and unearthed its unique features.

Jelena Guzina and Marko Djordjevic [8] explored the evolution of σ^70^ family of the factors by quantitative investigation of mix-and-matching in two canonical ECFfamily members (σ^E^ and σ^W^) and proposed an evolutionary anchored simple model which relates the size of σfactor regulon with the extent of mix-and-matching.

Aleksandra Chertkova and colleagues [9] employed *in silico* evolution of Drosophila gap gene regulatory sequence to demonstrate that elevated mutational pressure leads to produce regulatory sequence organizations with fewer, albeit on average stronger, functional transcription factor binding sites that support preservation of adjacent regulatory sequences.

The paper by Evgenii Konorov et al. [10] presents their insights into the genomics of urban populations of ants. Authors showed that the success of *Lasius niger* in urbanized areas may result from the expansion of the CYP9 cytochrome family, the diversification of DNA repair systems, and reduced odorant communication.

Finally, Ilyas Jetybaev et al. [11] studied short horned grasshoppers of the family Pamphagidae in the Anatolian region, and found two neo-XY sex chromosome systems, belonging to two different evolutionary lineages. They point at high density of species carrying neo-XY systems, and the different evolutionary stage for the two lineages found, one being older than the other.

The works presented above employ a variety of evolutionary analysis techniques, from karyotyping to genome sequencing and then to in silico evolution and to building kinetic models.

Returning back to the tenth anniversary BGRS\SB event, note that 2016’ thematic collection of the manuscripts is augmented by Special Issues covering various Satellite events, including already traditional Young Scientists School “Systems Biology and Bioinformatics” (SBB-2016) (http://conf.bionet.nsc.ru/sbb2016/en/). The CSGB-2016 Symposium on cognitive sciences has presented special issue at IEEE Xplore Digital Library (http://ieeexplore.ieee.org/xpl/mostRecentIssue.jsp?punumber=7587661). “Journal of Bioinformatics and Computational Biology” (http://www.worldscientific.com/worldscinet/jbcb) continues traditional special issues after BGRS\SB on mathematical problems of bioinformatics and biomedicine as well as “Journal of Integrative Bioinformatics” (http://journal.imbio.de/). Experimental research manuscripts fallen into the scope of BGRS\SB2016 have been collated in Special Issue of “Molecular Biology” (Springer) (http://www.springer.com/life+sciences/journal/11008) that will be published in 2017. In addition, special issue on bioinformatics and evolutionary biology is published at the “Vavilov Journal of Selection and Breeding” (http://www.bionet.nsc.ru/vogis).

The BGRS\SB-2016 Proceedings including “Evolutionary bioinformatics” section are available at the multi-conference web-site: http://www.bionet.nsc.ru/files/2016/conference/BGRS2016.pdf.

Next BGRS conference will take place in 2018. Welcome to Novosibirsk!
